# Decline of depressive symptoms in Europe: differential trends across the lifespan

**DOI:** 10.1007/s00127-020-01979-6

**Published:** 2020-11-12

**Authors:** Johannes Beller, Enrique Regidor, Lourdes Lostao, Alexander Miething, Christoph Kröger, Batoul Safieddine, Fabian Tetzlaff, Stefanie Sperlich, Siegfried Geyer

**Affiliations:** 1 Medical Sociology Unit, Hannover Medical School, Center for Public Health and Health Care, Carl-Neuberg-Street 1, 30625 Hannover, Germany; 2 Department of Public Health and Maternal and Child Health, Complutense University of Madrid, Madrid, Spain; 3 CIBER Epidemiología y Salud Pública (CIBERESP), Madrid, Spain; 4 Department of Medical Sociology, Public University of Navarre, Pamplona, Spain; 5 Department of Public Health Sciences, Stockholm University, Stockholm, Sweden; 6 Department of Clinical Psychology and Psychotherapy, Institute of Psychology, University of Hildesheim, Hildesheim, Germany

**Keywords:** Depression, Mental health, Trend, Population, Compression of morbidity

## Abstract

**Purpose:**

We examined changes in the burden of depressive symptoms between 2006 and 2014 in 18 European countries across different age groups.

**Methods:**

We used population-based data drawn from the European Social Survey (*N* = 64.683, 54% female, age 14–90 years) covering 18 countries (Austria, Belgium, Denmark, Estonia, Finland, France, Germany, Great Britain, Hungary, Ireland, The Netherlands, Norway, Poland, Portugal, Slovenia, Spain, Sweden, Switzerland) from 2006 to 2014. Depressive symptoms were measured via the CES-D 8. Generalized additive models, multilevel regression, and linear regression analyses were conducted.

**Results:**

We found a general decline in CES-D 8 scale scores in 2014 as compared with 2006, with only few exceptions in some countries. This decline was most strongly pronounced in older adults, less strongly in middle-aged adults, and least in young adults. Including education, health and income partially explained the decline in older but not younger or middle-aged adults.

**Conclusions:**

Burden of depressive symptoms decreased in most European countries between 2006 and 2014. However, the decline in depressive symptoms differed across age groups and was most strongly pronounced in older adults and least in younger adults. Future studies should investigate the mechanisms that contribute to these overall and differential changes over time in depressive symptoms.

## Introduction

Depression is one of the most common and severe mental impairments. It is estimated that about 264 million people worldwide are affected [32]. Besides being a serious disorder itself, depression is also a major predictor of other health-related outcomes like cardiovascular events, treatment non-compliance, musculoskeletal pain, absenteeism, dementia, and suicide [16, 20, 21, 45]. Accordingly, the Global Burden of Disease Study of the World Health Organization estimates that depression is one of the leading causes of disability [34, 54].

The general term depression encompasses a spectrum, ranging from single depressive symptoms, through subthreshold forms of depressive disorder, to severe recurrent major depressive disorder [2, 35]. It is increasingly recognized that even single symptoms of depression are of great public health importance, because even in the absence of a major depressive disorder, they could be associated with significant morbidity. For example, chronic feelings of loneliness have been described as being equally important to risk factors such as obesity and smoking [30], and have been found to predict mortality as well as impaired physical, functional, cognitive and mental health [5, 8, 9, 19]. Thus, due to its high prevalence and its far-reaching individual and societal consequences, depression is of major public health importance.

Therefore, there is a need to study cross-temporal differences in depressive symptoms. Several studies have analysed trends in depression, mostly focusing on specific age-groups. For example, Keyes et al. [36] recently analysed trends in depression of students of the 8th, 10th, and 12th grade from 1991 to 2018. Depression was measured as a continuous construct via self-report as a sum score of four depressive symptoms. The authors found that depressive symptoms increased among teens in the United States over time, especially among girls. Similarly, another study analysed cross-temporal differences of a sum-score of self-reported depressive symptoms in Icelandic adolescents and also found increasing levels of depression in girls [52]. In a similar age range, Mojtabai et al. [48] analysed trends in the prevalence of depression using a short structured interview among youth aged 12–25. They found an increase, especially among adolescents. Zivin et al. [59] analysed trends of depression among older adults in the US. Using prevalence estimates based on the eight-item Center for Epidemiologic Studies Depression Scale (CES-D 8), they found that depressive symptoms decreased over time in US older adults. Similarly, Sullivan et al. [51] used a modified version of the CES-D 8, analysed a continuous depressive symptom burden score as well as a dichotomous cut-off and found that depressive burden of older adults decreased across birth cohorts. Hence, evidence on trends in depression mostly originates from the US and focuses on specific age groups.

Only few studies used the whole age range, including youth, young adults, middle-aged adults and older adults, and examined whether there were significantly differential trends across the lifespan (i.e., for people of different ages, from young to older adults). In one of these studies, Weinberger et al. [56] examined trends in the depression prevalence in the US. The authors found that depression prevalence significantly increased across time, but that this increase significantly differed between age groups, with depression in the youngest increasing the most strongly. In another study, Bretschneider et al. [15] examined trends in the depression prevalence in Germany. In their study, the depression prevalence was found to be relatively constant overall. However, the authors also found a shift in the age distribution in women, with significant increases in the depression prevalence among the young age group (age 18–34), no significant change in the middle-aged age group (age 35–49) and a significant decrease in the old age group (age 50–65). All studies point towards the need to further study cross-temporal differences in depression, considering the whole lifespan and using multi-national samples.

From a theoretical point of view, several theories have been developed to describe the general morbidity development at the population level. Among them, Fries [22] suggested that over time serious morbidity will occur ever later in the life course, such that there is a compression of morbidity towards the end of life. Contradicting this position, Gruenberg [27] and Kramer [39] suggested that there will be increasing morbidity, especially concerning mental disorders and physical chronic conditions. Whereas numerous studies have focused on physical conditions such as functional limitations, evidence regarding mental disorders is missing [11, 23].

The current study aims to address these issues. It contributes to the literature by examining changes in depressive symptoms between 2006 and 2014 in several European countries (Austria, Belgium, Denmark, Estonia, Finland, France, Germany, Great Britain, Hungary, Ireland, The Netherlands, Norway, Poland, Portugal, Slovenia, Spain, Sweden, Switzerland) by covering the age groups from youth to old age (age range: 14–90; *N* = 64,683). Thereby the current study clarifies:

- whether the depressive symptom burden has changed in Europe between 2006 and 2014;
- whether the burden of depressive symptoms differ across age groups (14–90 years);
- whether these potential changes in depressive symptoms can be generalized across countries in Europe; and
- how these potential differences might be explained.

## Materials and methods

### Sample

Data were drawn from the public release of the cumulative European Social Survey (ESS) that aims to provide comparative data on attitudes, beliefs and behaviour patterns of the various populations in Europe. The ESS also includes rotating modules, which are dedicated to specific themes that are sometimes repeated in later survey rounds. We used data from the eighteen countries (Austria, Belgium, Denmark, Estonia, Finland, France, Germany, Great Britain, Hungary, Ireland, The Netherlands, Norway, Poland, Portugal, Slovenia, Spain, Sweden, Switzerland) that participated in the 2006 and 2014 waves of the ESS, which included rotating modules relating to personal well-being and health. To date, only the 2006 and 2014 waves incorporated measures of depressive symptoms, and as such only these two waves could be included in the current study. The ESS provides population-based cross-sectional samples of non-institutionalized participants aged 14 years and older with the interviews conducted face-to-face at the respondent’s place of residence. Thus, at both time points, population-based cross-sectional samples of first-time responders were obtained. Response rates were between 46 and 73% in 2006 per country (Austria: 65%, Belgium: 61%, Denmark: 51%, Estonia: 65%, Finland: 64%, France: 46%, Germany: 55%, Great Britain: 55%, Hungary: 66%, Ireland: 57%, The Netherlands: 60%, Norway: 66%, Poland: 70%, Portugal: 73%, Slovenia: 65%, Spain: 66%, Sweden: 66%, Switzerland: 52%) and between 31 and 68% in 2014 per country (Austria: 52%, Belgium: 57%, Denmark: 52%, Estonia: 60%, Finland: 63%, France: 51%, Germany: 31%, Great Britain: 44%, Hungary: 53%, Ireland: 61%, The Netherlands: 59%, Norway: 54%, Poland: 66%, Portugal: 43%, Slovenia: 53%, Spain: 68%, Sweden: 50%, Switzerland: 53%). All procedures were in accordance with the ethical standards of the institutional research committee and with the 1964 Helsinki declaration and its later amendments. After excluding participants with missing values listwise (about 4.3% of the sample), a final sample with *N* = 64,683 participants resulted (*N*_2006_ = 32,578; *N*_2014_ = 32,105).

### Measures

Depressive symptoms were measured with the 8-item version of the Centre of Epidemiologic Studies Depression Scale (CES-D 8) in both 2006 and 2014. Multiple studies have demonstrated the scale’s validity and reliability to measure depressive symptoms in different cultures and throughout the lifespan (e.g., [33, 37, 38, 55]). The CES-D 8 measures the frequency of depressive symptoms in the week prior to the interview. Participants were asked whether they (1) “felt depressed”, (2) “felt lonely”, (3) “felt sad”, (4, reverse scored) “were happy”, (5, reverse scored) “enjoyed life”, (6) “felt everything was an effort”, (7) “had restless sleep”, and (8) “could not get going”. Participants could choose to respond with one of four response options ranging from “none or almost none of the time” (score 0) to “all or almost all of the time” (score 3). In accordance with psychometric evidence, a dimensional total depressive symptoms score was calculated as the sum of all responses ranging from 0 to 24 [41]. Additionally, it was investigated whether the results are robust to the analysis of a dichotomous depression score via cut-off of larger than 9. In the current study, reliability of the CES-D 8 sum score was acceptable (2006: Cronbach’s *α* = 0.83, 2014: Cronbach’s *α* = 0.82). In addition to CES-D 8, age, sex, education (measured via the years of full-time education completed) and income and health were assessed. Income was measured via inquiring whether the participants had serious difficulties to cope with the present household income [score = 1], whether they had difficulties coping with the current household income [score = 2], whether they could cope with the present household income [score = 3], or whether they could live comfortably with their household income [score = 4]. Finally, current health status was operationalized by asking about participant’s self-rated health with answer options ranging from “very bad” [score = 1], “bad” [score = 2], “fair” [score = 3] and “good” [score = 4] to “very good” [score = 5].

### Data analysis

First, descriptive statistics of all variables across and within countries and the two time periods are reported in the results section. Then bivariate generalized additive model regression analyses were conducted to graphically examine how the CES-D 8 score changes across age. Generalized additive model regressions might be favourably used in the current study, because they are able to depict highly non-linear relationships between variables by fitting several non-linear regression models to multiple localized subsets of the data. Then, to model the average change in depressive symptoms over countries, multilevel regression analyses were applied, controlling for age only. In addition, to estimate how much of the average change in depressive symptoms can be explained by our covariates, we included our full set of covariates (age, sex, education and income) as quantitative covariates in additional multilevel regressions. All participant characteristics were included on level 1. Country was used as the level 2 grouping factor to account for cross-national differences in depression via varying intercepts. To examine age-specific changes between groups, these multilevel regression analyses were also stratified in three age groups (young adults: 14–39; middle-aged adults: 40–64; older adults: 65 +), as has been similarly done in the literature [15]. Differences in regression coefficients between age-groups were examined by *z*-test [17]. Additionally, to better explain these changes in depressive symptom burden over time, education, income and health were included as covariates in further multilevel regression analyses. Finally, to examine the change in depressive symptoms specific for each country, age-stratified linear regression analyses within countries using the country-specific data were conducted. All inferential statistical methods used the design weights provided by the European Social Survey. All statistical analyses were performed with R.

## Results

Overall, participants were on average 47.46 (standard deviation [SD] = 18.29) years old in 2006 and 49.26 (SD = 18.48) years old in 2014, with 54% being female in 2006 and 52% in 2014. Participants had on average 12.29 (SD = 4.25) years of education in 2006 and 12.96 (SD = 4.06) years of education in 2014. In 2006 and 2014, participants reported that they could cope with their current income (2006: mean [*M*] = 3.12, SD = 0.80; 2014: *M* = 3.12, SD = 0.81, on a scale of 1–4) and that they had good health (2006: *M* = 3.81, SD = 0.90; 2014: *M* = 3.82, SD = 0.90, on a scale of 1–5). On average, the sum score of depressive symptoms declined in 2014 (2006: *M* = 5.72, SD = 4.10; 2014: *M* = 5.19, SD = 3.98).

**Fig. 1:**
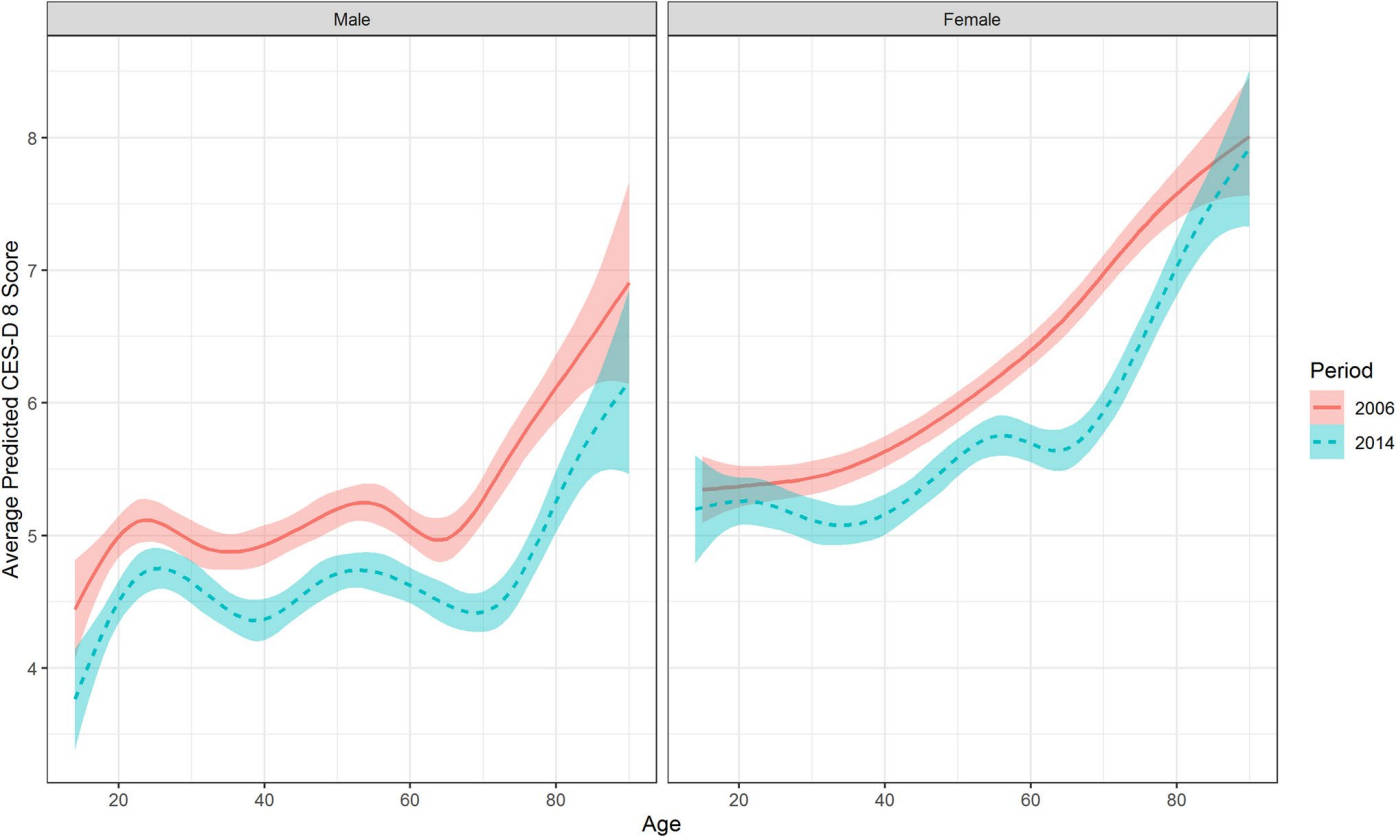
Average CES-D 8 scores across age between 2006 and 2014 predicted via generalized additive models (shaded areas represent 95% confidence intervals)

Next, we examined differential cross-temporal changes in depressive symptom burden across the lifespan using non-linear generalized additive models. As can be seen in Fig. 1, although depressive symptoms tended to increase with increasing age, a highly non-linear association between both variables emerged. Depressive symptoms appeared to decrease from 2006 to 2014 for the whole age range. The decline in the sum score for depressive symptoms appeared to be strongest in older adults. These effects were in general replicated with single depressive symptoms, although it appears that declines were smaller or even partly reversed into increases in the case of younger adults regarding fatigue, loneliness, and sleep problems Appendix Fig. 3. The results are also replicated when using the proposed cut-off (sum score > 9) to denote a depression prevalence Appendix Fig. 4.

These descriptive differences of decreasing depressive symptoms were confirmed by the age-stratified multilevel regression analyses as depicted in Table 1. Depressive symptoms decreased significantly in women and men and for all age-groups, but the decline was most pronounced in older adults. Pairwise *z* tests indicated that this decline was indeed significantly strongest in older adults as compared to young- and middle-aged adults both in women and men (all *p* values < 0.05). As seen in Table 1, when including the covariates the decline in depressive symptoms was not substantially reduced in younger and middle-aged adults. However, after controlling for education, health and income, the decline was substantially reduced in older men (− 0.813 vs. − 0.676), and women (− 0.840 vs. − 0.489).

**Table 1:** Changes in depressive symptoms (CES-D 8 scores) from 2006 to 2014 in Europe via multilevel regression analyses without and with a full set of covariates (*N* = 64,683)

	Age: 14–39						Age: 40–65						Age: 66 +					
	Model 1			Model 2			Model 1			Model 2			Model 1			Model 2		
	*b*	χ^2^	*p*	*b*	χ^2^	*p*	*b*	χ^2^	*p*	*b*	χ^2^	*p*	*b*	χ^2^	*p*	*b*	χ^2^	*p*
Women (*N* = 34,265)																		
Time	− 0.312	19.95	< 0.001	− 0.270	17.46	< 0.001	− 0.424	39.55	< 0.001	− 0.380	40.42	< 0.001	− 0.840	74.86	< 0.001	− 0.489	32.43	< 0.001
Age	0.003	0.46	0.496	− 0.022	21.15	< 0.001	0.030	39.29	< 0.001	− 0.005	1.32	0.250	− 0.087	133.92	< 0.001	0.032	22.45	< 0.001
Health				− 1.574	1339.96	< 0.001				− 1.741	2250.09	< 0.001				− 2.070	1653.93	< 0.001
Education				− 0.025	6.28	0.012				− 0.022	7.68	0.006				− 0.020	2.72	0.099
Income				− 0.829	360.67	< 0.001				− 1.245	954.52	< 0.001				− 1.081	329.12	< 0.001
Men (*N* = 30,418)																		
Time	− 0.438	45.84	< 0.001	− 0.397	43.41	< 0.001	− 0.492	60.40	< 0.001	− 0.441	61.01	< 0.001	− 0.813	74.54	< 0.001	− 0.676	65.14	< 0.001
Age	0.006	1.65	0.199	− 0.021	23.33	< 0.001	0.010	5.46	0.019	− 0.016	14.87	< 0.001	− 0.077	99.81	< 0.001	0.045	42.73	< 0.001
Health				− 1.432	1156.32	< 0.001				− 1.482	1681.87	< 0.001				− 1.750	1209.65	< 0.001
Education				0.013	1.92	0.166				0.003	0.169	0.681				− 0.015	2.14	0.144
Income				− 0.798	368.15	< 0.001				− 1.180	865.08	< 0.001				− 0.905	206.00	< 0.001

Time = adjusted difference in CES-D 8 scores between 2006 and 2014; *b* = regression coefficient; χ^2^ = χ^2^ test statistic; *p* = *p* value

**Fig. 2:**
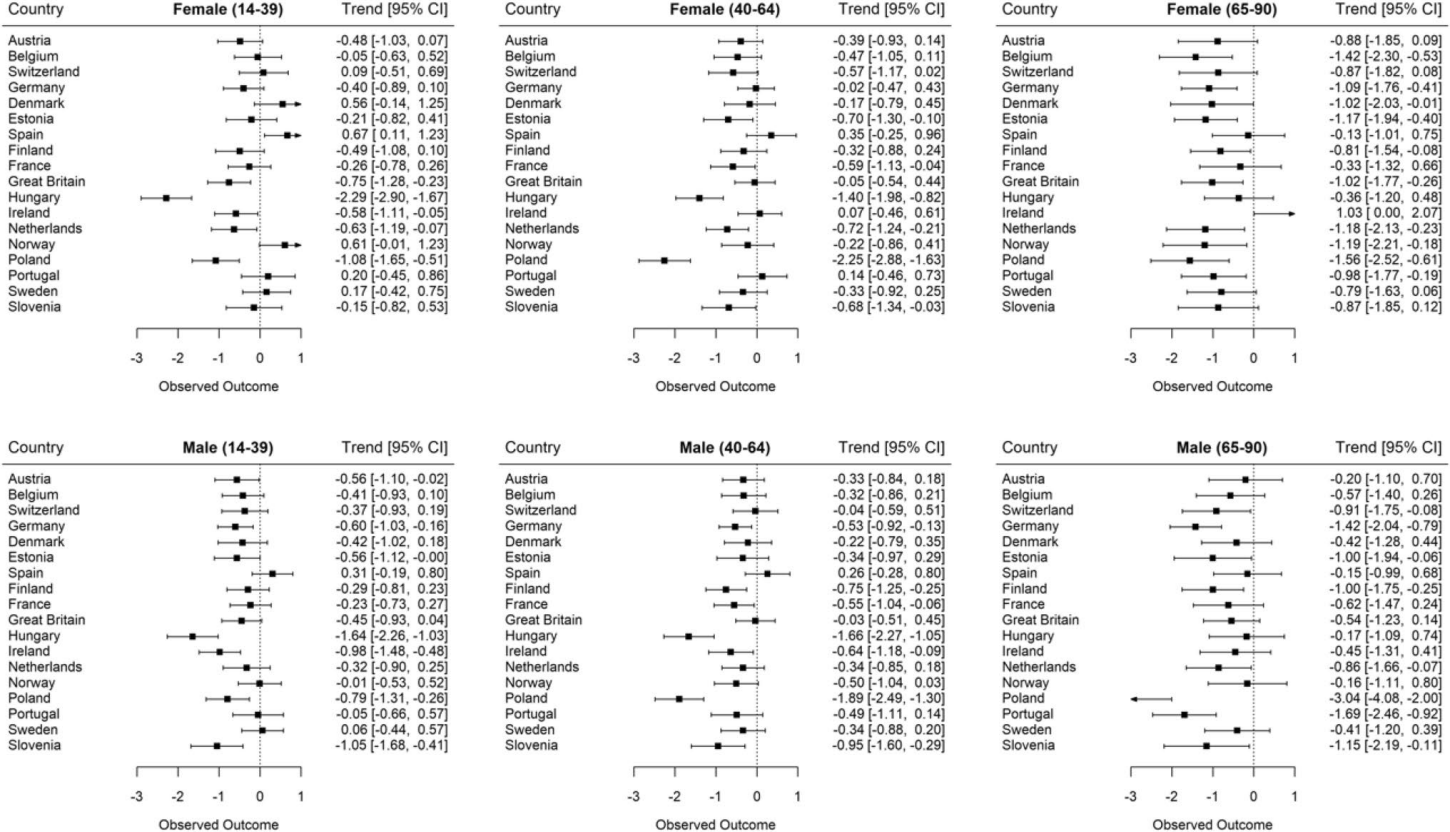
Changes in depressive symptoms (adjusted mean CES-D 8 score differences with 95% confidence intervals) between 2006 and 2014 across age-groups and within countries controlled for age

Finally, we examined the generalizability of the results by investigating changes in depressive symptom burden within countries, as visualized in Fig. 2 and described in Appendix Tables 2 and 3. Strongest decreases in depressive symptoms were generally observed in Hungary, Poland and Slovenia, whereas the smallest decreases, and among younger adults partly even increases, in depressive symptoms were generally observed in Spain, Norway and Denmark. However, the country-specific results were similar to the overall results reported in the previous paragraph. Except for Spain and, in the case of women, Denmark and Norway, younger and middle-aged adults’ depressive symptoms tended to decline. And, except for Ireland, older adults’ depressive symptoms also declined but to a greater extent. These general changes were replicated when analysing a specific youth age group (age 14–24, Appendix Fig. 5), although it appears that the country-specific increases in depressive symptoms tended to be stronger in this age bracket. Thus, the country-specific results supported the overall multilevel analyses in that depressive symptoms decreased most strongly in older adults.

## Discussion

We examined cross-temporal changes in depressive symptoms between 2006 and 2014 for multiple countries and different age groups. We found that there was a general decline in depression across time for all age-groups. The strength of this decline differed across the age range: consistent across both sexes and for all countries analysed, we found that depressive symptoms declined most in older adults and less strongly in younger and middle-aged adults.

Our results both contradict and confirm previous studies. Numerous studies had suggested that depression increased in younger adults (e.g., [36, 48, 52]). We found this not to be the case in general. Although young adults’ depression scores increased in some countries, in the majority of countries depressive symptoms actually decreased. Contrarily to these trends, some studies had suggested that depression decreased in older adults (e.g., [51, 29]). Supporting these studies, we also found that depression scores decreased strongly for older adults in most countries. Directly comparing these effects in different age groups, we found that there were indeed significantly differential cross-temporal changes in depression across the lifespan, with older adults improving the most. Thus, going beyond the previous literature, the current study suggests (a) that the pattern of cross-temporal changes in depression differs across age, (b) that these changes can be generalized across European countries for older and middle-aged but not for younger adults and (c) that the decreases in middle-aged and older adults’ depression symptoms burden might largely be explained by predictors relating to general health and socio-economic status, but that these predictors cannot explain the observed results in young adults.

However, several limitations have to be taken into account when interpreting our results. The sample did not include institutionalized older adults and thus likely underestimates the true level of depression in the population. Additionally, we only compared two time points, instead of several. Changes in the level of depression might also be partly explained by changing response rates between time points. It might be the case that potential participants with high depressive symptom burden have become less likely to participate in research. If this was true, then the observed differences would not only be due to changes in the population level of depressive symptom burden but also due to changes in participation rates between time points. Thus, selective participation and systematic dropout are important topics that need to be analysed in future studies. Similarly, although we analysed population-based data of multiple countries, these were only European countries and other continents were not represented [28], and levels of depressive symptom burden and changes in depressive symptom burden might differ in other countries. Of course, as the current study still strongly suggests that cross-temporal changes and geographical variations in depressive symptom burden exist, more research on this topic is needed.

We also analysed only a self-report measure of depression, the CES-D 8. As self-reported measures might also be susceptible to biases [44], future studies should investigate differential cross-temporal changes for different age-groups using more objective indicators of depression, like interview-based symptom scores. In a similar vein some of our covariates to explain depression differences, such as income and education, might be sub-optimal indicators in youth and younger adults as compared to middle-aged and older adults. Thus, future studies should try to examine additional predictors of cross-temporal changes in depressive symptom burden.

It also remains to be investigated why cross-temporal changes in some countries diverged from the general results. Contrarily to general trends, Spanish younger and middle-aged adults seemed to exhibit increasing depression scores. Some studies have shown an increase in poor mental health, using the Goldberg 12-item scale questionnaire, between 2006 and 2011 in men under 65, but not in women [4, 53]. This finding has been attributed to the impact of the economic crisis, since the increase was greater in unemployed men [4, 53]. Similarly, although not as statistically significant, young adults’ depression scores in the Nordic countries seemed to decrease less than in other countries or even increased. Several studies speculated about rising socioeconomic inequalities and perceived stress as possible explanation for this phenomenon [1, 57]. For example, in the Swedish context it has been found that major changes in the school system were accompanied by lower school achievements and lower employment rates, which then might explain the less favorable developments in mental health among younger adults from Nordic countries like Sweden [14]. Finally, in contrast to older adults from other countries, older female adults in Ireland were found to also significantly increase in depression scores. This seems especially concerning as studies have found high rates of depression in women in Ireland, in general [43]—however, empirical evidence that might explain this finding is lacking. For example, cross-national variations in depressive symptom burden might also be partly due to differing response rates. However, contrarily to this preposition, most countries showed declines in depressive symptom burden irrespective of the respective changes in response rates. Similarly, increases in depressive symptom burden partly occurred in Denmark, Spain and Sweden, although response rates changed differentially between these countries. Still, future studies are needed that provide empirical explanations for the observed differences across countries. Finally, some studies have pointed to the existence of different symptom profiles across the lifespan [47]. Future studies might examine whether, in addition to additive changes in the symptom severity, there are also changes in depression profiles across the life span over time.

The result that older adults have benefitted the most is important from a demographic perspective. Increases in life expectancy and decreases in fertility are leading to an ever increasing share of older adults in the general population [12]. As such, the finding that morbidity in the form of depressive symptoms in older adults decreased strongest opposes Greenberg’s expansion of morbidity hypothesis, which states that over time old age will be increasingly associated with higher levels of morbidity [27]. At the same time, these results support Fries’ morbidity compression hypothesis that morbidity at the end of life can be managed and appears to be reducing [22].

The finding that younger adults’ depression scores decreased the least and even appeared to be increasing in some countries and regarding some depressive symptoms is alarming from a public health perspective. Depressive symptoms are seen as relatively stable throughout the life course [42, 49]. As such, if some of today’s young adults start off with more depressive symptoms than previous generations, then they are also likely to experience significantly more depressive symptoms as they age. This would not only impede their physical, mental and cognitive health, but it would also necessitate a greater burden on health care systems as well. Thus, more research is needed to clarify whether younger age cohorts actually increasingly experience depressive symptoms, for example using age-period-cohort analysis [5–7, 58]. Additionally, special prevention efforts should be targeted at younger adults. However, to effectively improve health of these groups, knowledge about the reasons for their health impediment is indispensable.

There are several possible explanations for these cross-temporal changes. Medical and societal advances might have improved treatment availability and treatment effectiveness: Studies have found that help-seeking behaviour for depressive symptoms and the effectiveness of treatments for depressive symptoms has increased over time [13, 29, 40]. This might also explain the increasing frequency of depression diagnoses [31, 50]. As more people with depressive symptoms seek help, the observed administrative prevalence of depression increases and the depressive symptom burden in the population decreases. As such people might be treated earlier and more effectively for depressive symptoms over time [18].

Additionally, we examined some potential predictors of depressive symptom burden changes in the current study. It was found that the inclusion of education, income and health substantially decreased the decline in older adults. This suggests that a combination of the expansion of education, the decrease in poverty, and the improvement of general health have likely contributed to the substantial decrease of depressive symptoms in older adults [23, 46]. At the same time, including these covariates could not substantially explain declines in young and middle-aged adults. Thus, further research must examine age-specific risk factors for depression that might explain these differential effects, such as problematic social media use, or changes in other lifestyle factors [3, 25, 26]. Another methodological explanation is that cognitive biases might have unduly influenced self-reports. Perhaps, in accordance with an anchor effect, the self-perceived threshold of what it means to experience depressive symptoms has increased over time in older adults, such that the reporting but not the level of depression has decreased [24]. Lastly, however, future studies must empirically analyse the origin of these changes and why they appear especially strong in older adults but are less pronounced in younger age cohorts.

Summing up, we investigated time differences in depressive symptoms in multiple European countries and analysed whether these cross-temporal changes differed across age. Using population-based data of Europeans from 18 countries, we found that there was a general decline in depressive symptoms. However, time differences in depression differed significantly across the lifespan: with few exceptions, depressive symptoms declined most strongly in older adults and least in younger adults. Future studies should investigate the mechanisms that contribute to these overall and differential trends across the lifespan.

## Figures and Tables

**Fig. 3:**
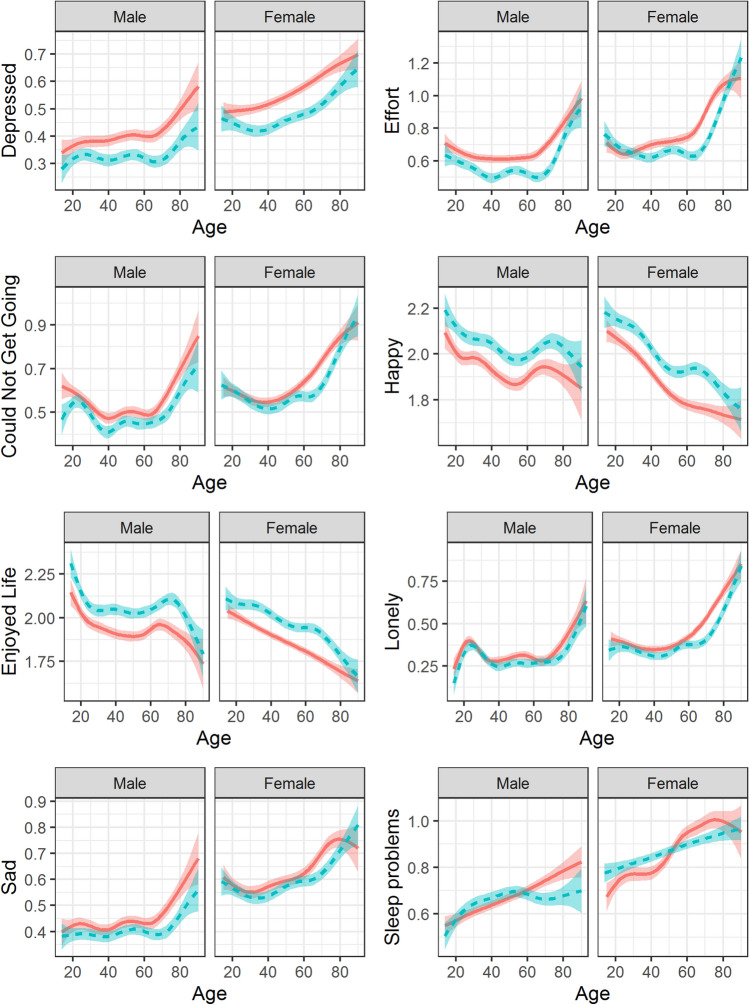
Average depressive symptom item scores (CES-D 8 items; range: 0–3) across age between 2006 (solid line) and 2014 (dashed line) predicted via generalized additive models (shaded areas represent 95% confidence intervals)

**Fig. 4:**
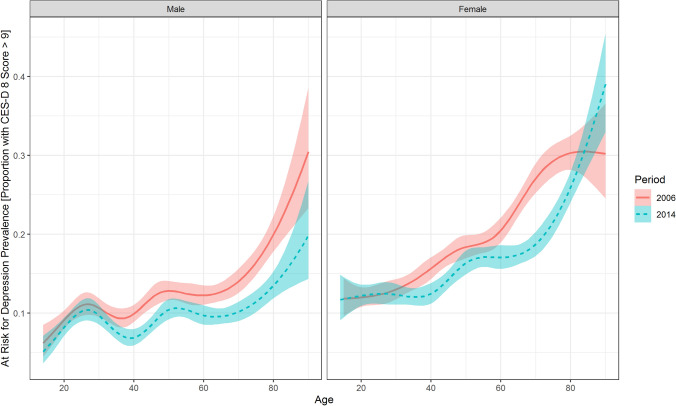
Prevalence of at-risk depression via cut-off (CES-D 8 score > 9) across age between 2006 and 2014 predicted via generalized additive models (shaded areas represent 95% confidence intervals)

**Fig. 5:**
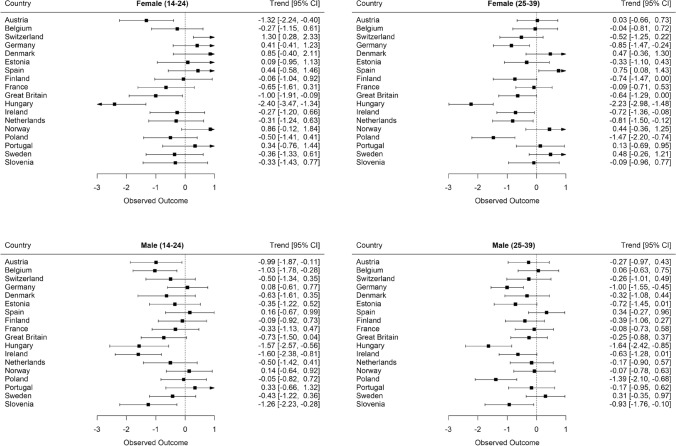
Changes in depressive symptoms (adjusted mean CES-D 8 score differences with 95% confidence intervals) from 2006 to 2014 in youth (age 14–24) and young adults (age 25–39) within countries adjusted for age

## Appendix

See Tables 2 and 3, Figs. 3, 4, and 5

**Table 2:** Mean CES-D 8 scores across age groups, time periods and countries in women

Country	Age 14–39						Age 40–65						Age 66 +					
	2006			2014			2006			2014			2006			2014		
	*N*	*M*	95% CI	*N*	*M*	95% CI	*N*	*M*	95% CI	*N*	*M*	95% CI	*N*	*M*	95% CI	*N*	*M*	95% CI
Austria	449	5.08	[4.73; 5.44]	313	4.41	[4.04; 4.77]	564	5.56	[5.24; 5.89]	408	5.06	[4.68; 5.44]	164	7.48	[6.77; 8.19]	313	4.41	[4.04; 4.77]
Belgium	350	5.67	[5.26; 6.09]	311	5.62	[5.19; 6.04]	414	6.07	[5.63; 6.50]	396	5.52	[5.10; 5.95]	180	6.86	[6.18; 7.53]	311	5.62	[5.19; 6.04]
Switzerland	309	4.61	[4.25; 4.96]	281	4.53	[4.11; 4.95]	447	5.04	[4.72; 5.36]	327	4.33	[3.93; 4.73]	204	5.70	[5.17; 6.23]	281	4.53	[4.11; 4.95]
Germany	442	5.87	[5.54; 6.19]	453	5.53	[5.19; 5.87]	664	6.22	[5.93; 6.50]	684	6.05	[5.77; 6.33]	291	7.73	[7.26; 8.21]	453	5.53	[5.19; 5.87]
Denmark	232	4.79	[4.40; 5.19]	227	5.40	[4.91; 5.89]	355	5.09	[4.71; 5.47]	343	4.86	[4.45; 5.27]	131	5.05	[4.43; 5.66]	227	5.40	[4.91; 5.89]
Estonia	261	5.92	[5.51; 6.32]	346	5.73	[5.37; 6.10]	308	7.09	[6.64; 7.54]	486	6.48	[6.12; 6.84]	183	8.72	[8.02; 9.42]	346	5.73	[5.37; 6.10]
Spain	376	5.31	[4.92; 5.69]	318	5.97	[5.49; 6.46]	335	6.21	[5.73; 6.68]	390	6.61	[6.14; 7.08]	184	8.38	[7.61; 9.15]	318	5.97	[5.49; 6.46]
Finland	321	4.99	[4.61; 5.36]	306	4.47	[4.11; 4.83]	419	4.88	[4.55; 5.20]	448	4.52	[4.21; 4.83]	229	5.63	[5.16; 6.10]	306	4.47	[4.11; 4.83]
France	368	5.74	[5.29; 6.20]	325	5.61	[5.18; 6.04]	476	6.38	[5.95; 6.81]	420	5.89	[5.47; 6.32]	194	6.77	[6.14; 7.40]	325	5.61	[5.18; 6.04]
Great Britain	433	6.13	[5.74; 6.51]	343	5.58	[5.15; 6.01]	527	6.06	[5.68; 6.45]	544	5.89	[5.50; 6.27]	317	6.34	[5.87; 6.82]	343	5.58	[5.15; 6.01]
Hungary	246	7.61	[7.03; 8.18]	287	5.37	[4.96; 5.77]	353	8.92	[8.43; 9.40]	414	7.31	[6.90; 7.72]	229	10.63	[9.88; 11.39]	287	5.37	[4.96; 5.77]
Ireland	344	5.04	[4.65; 5.43]	418	4.37	[4.03; 4.72]	345	4.70	[4.32; 5.07]	559	4.77	[4.44; 5.10]	138	4.75	[4.16; 5.35]	418	4.37	[4.03; 4.72]
The Netherlands	334	5.36	[5.00; 5.72]	321	4.63	[4.29; 4.97]	466	5.54	[5.17; 5.91]	497	4.91	[4.57; 5.26]	200	7.21	[6.66; 7.76]	321	4.63	[4.29; 4.97]
Norway	334	4.61	[4.25; 4.98]	241	5.25	[4.79; 5.72]	376	4.25	[3.97; 4.53]	294	4.01	[3.65; 4.38]	140	4.76	[4.16; 5.37]	241	5.25	[4.79; 5.72]
Poland	368	5.31	[4.88; 5.73]	311	4.35	[3.88; 4.83]	343	8.13	[7.59; 8.66]	346	6.03	[5.48; 6.58]	128	9.97	[9.01; 10.93]	311	4.35	[3.88; 4.83]
Portugal	369	6.46	[6.05; 6.86]	180	6.48	[5.93; 7.04]	548	8.57	[8.18; 8.96]	278	8.24	[7.64; 8.85]	366	10.34	[9.85; 10.83]	180	6.48	[5.93; 7.04]
Sweden	338	5.49	[5.05; 5.93]	295	5.63	[5.15; 6.10]	429	5.25	[4.87; 5.64]	361	4.95	[4.58; 5.32]	178	5.94	[5.30; 6.58]	295	5.63	[5.15; 6.10]
Slovenia	269	5.11	[4.73; 5.50]	213	4.92	[4.42; 5.42]	338	5.73	[5.31; 6.16]	284	5.15	[4.72; 5.58]	152	8.41	[7.64; 9.17]	213	4.92	[4.42; 5.42]

*N* sample size,* M* mean, *95% CI* 95% confidence interval

**Table 3:** Mean CES-D 8 scores across age groups, time periods and countries in men

Country	Age 14–39						Age 40–65						Age 66 +					
	2006			2014			2006			2014			2006			2014		
	*N*	*M*	95% CI	*N*	*M*	95% CI	*N*	*M*	95% CI	*N*	*M*	95% CI	*N*	*M*	95% CI	*N*	*M*	95% CI
Austria	392	4.79	[4.46; 5.11]	269	4.06	[3.67; 4.45]	471	5.34	[4.99; 5.68]	374	4.78	[4.42; 5.15]	138	5.8	[5.07; 6.54]	177	5.07	[4.53; 5.61]
Belgium	323	4.84	[4.48; 5.21]	343	4.44	[4.10; 4.78]	367	4.59	[4.19; 4.99]	387	4.3	[3.97; 4.63]	140	4.89	[4.2; 5.59]	153	4.35	[3.73; 4.96]
Switzerland	249	4.48	[4.12; 4.84]	275	3.97	[3.62; 4.33]	392	4.32	[3.99; 4.64]	319	4.17	[3.77; 4.57]	158	4.3	[3.8; 4.79]	160	3.33	[2.87; 3.78]
Germany	461	5.57	[5.25; 5.88]	450	5.04	[4.73; 5.35]	649	5.54	[5.28; 5.79]	739	4.96	[4.72; 5.20]	238	5.96	[5.48; 6.45]	320	4.53	[4.17; 4.89]
Denmark	212	5.02	[4.62; 5.43]	291	4.62	[4.23; 5.02]	369	4.33	[4.03; 4.62]	304	4.13	[3.77; 4.50]	116	3.91	[3.28; 4.53]	164	3.43	[2.89; 3.98]
Estonia	246	5.76	[5.35; 6.17]	322	5.20	[4.82; 5.58]	229	6.39	[5.89; 6.88]	342	6.08	[5.66; 6.51]	100	7.52	[6.65; 8.39]	139	6.6	[5.98; 7.23]
Spain	379	4.60	[4.26; 4.93]	336	4.92	[4.53; 5.31]	318	4.79	[4.34; 5.23]	428	5.12	[4.75; 5.49]	137	5.81	[5.04; 6.58]	166	5.57	[4.89; 6.25]
Finland	338	4.55	[4.27; 4.83]	310	4.27	[3.93; 4.60]	409	4.83	[4.50; 5.15]	470	4.13	[3.87; 4.39]	154	5.27	[4.72; 5.81]	221	4.14	[3.73; 4.54]
France	328	4.80	[4.42; 5.18]	295	4.57	[4.20; 4.94]	440	4.87	[4.52; 5.22]	414	4.56	[4.23; 4.88]	152	5.13	[4.47; 5.79]	186	4.52	[3.99; 5.05]
Great Britain	373	5.53	[5.15; 5.91]	274	5.03	[4.55; 5.51]	459	5.24	[4.88; 5.61]	430	5.16	[4.77; 5.54]	220	5.49	[4.94; 6.04]	290	4.77	[4.34; 5.21]
Hungary	204	6.55	[5.98; 7.12]	227	5.03	[4.56; 5.49]	254	8.82	[8.15; 9.49]	317	6.54	[6.10; 6.98]	128	9.23	[8.37; 10.08]	144	8.77	[8.03; 9.51]
Ireland	269	4.90	[4.47; 5.33]	349	3.98	[3.59; 4.38]	296	4.63	[4.21; 5.04]	459	4.12	[3.77; 4.47]	131	4.97	[4.33; 5.61]	241	4.78	[4.25; 5.31]
The Netherlands	305	4.56	[4.20; 4.93]	228	4.35	[3.88; 4.82]	393	4.59	[4.25; 4.93]	385	4.12	[3.75; 4.48]	153	5.12	[4.49; 5.76]	220	4.16	[3.68; 4.64]
Norway	350	4.24	[3.93; 4.55]	286	4.26	[3.93; 4.58]	429	3.94	[3.66; 4.23]	337	3.42	[3.13; 3.72]	107	3.71	[3.12; 4.3]	128	3.48	[2.99; 3.98]
Poland	360	4.59	[4.23; 4.94]	287	3.82	[3.39; 4.24]	323	6.79	[6.27; 7.30]	295	5.09	[4.57; 5.61]	88	8.35	[7.25; 9.46]	103	5.18	[4.34; 6.03]
Portugal	291	5.55	[5.14; 5.95]	177	5.37	[4.80; 5.94]	309	6.57	[6.13; 7.01]	213	5.96	[5.39; 6.53]	222	8.29	[7.77; 8.82]	165	6.5	[5.84; 7.15]
Sweden	390	4.51	[4.19; 4.83]	303	4.53	[4.15; 4.91]	396	4.38	[4.04; 4.73]	337	4.01	[3.62; 4.40]	140	4.41	[3.81; 5.01]	217	4.03	[3.63; 4.43]
Slovenia	265	4.97	[4.65; 5.30]	184	3.87	[3.46; 4.28]	271	5.27	[4.88; 5.67]	240	4.47	[4.02; 4.91]	89	6.29	[5.46; 7.12]	108	5.19	[4.41; 5.96]

*N* sample size,* M* mean, *95% CI* 95% confidence interval

.
